# Retraction Note: The quantum physiology of oxygen; from electrons to the evolution of redox signaling in the human brain

**DOI:** 10.1186/s42234-019-0026-y

**Published:** 2019-06-20

**Authors:** Damian Miles Bailey

**Affiliations:** University of South Wales, Pontypridd, UK

**Retraction to: Bioelectronic Medicine (2018) 4:13.**

**https://doi.org/10.1186/s42234-018-0014-7**

**Retraction Note**

This article (Bailey, 2018) has been retracted at the request of the author due to significant overlap with one of his other publications (Bailey, 2019) without proper citation. This article is considered redundant. The author agrees to this retraction.

## References

[CR1] Bailey DM (2018). The quantum physiology of oxygen; from electrons to the evolution of redox signaling in the human brain. Bioelectronic Med.

[CR2] Bailey DM (2019). Oxygen, evolution and redox signalling in the human brain; quantum in the quotidian. J Physiol.

